# Difficult conditions of the spastic upper limb

**DOI:** 10.1186/1753-6561-9-S3-A71

**Published:** 2015-05-19

**Authors:** Praveen Bhardwaj

**Affiliations:** 1 Ganga Hospital, Coimbatore, 641043, India

## 

“Difficult” is a very relative terminology and “difficult” to generalize. The various operative procedures done in patients with spastic hand are not technically demanding but assessing and decision making is the real challenge in these patients. There are some difficulties, which I personally encounter while assessing these patients and they have a bearing on the decision making. There are some other challenges which are characteristic of the disease, which are not correctable and they remain “difficult”. Some uncommon presentations can at times pose 'difficulty'. We can divide the difficult conditions into three categories:

## Assessment and decision-making

In mild cases of spasticity, it requires detailed, repeated assessment of the patient to understand the problem with the hand and the need for intervention. Dynamic flexion deformity of the wrist is a common but relatively neglected category. The patient on examination would be having good flexion and extension of the wrist and fingers but they would complain of inability to use the hand for day to day activities. While assessing them at work one would note that their wrist flexes excessively when they try to open up the fingers and also flexes when they try to hold objects tightly and hence their grasp on things is weaker. On clinical examination one can note hyperactivity of FCU when the patient is asked to extend the fingers and also out of phase activity of FCU when power grasp is made. Such patients do well with FCU tenotomy or FCU to ECRB tendon transfer in less tension depending on the power of the wrist extensors.

Swan neck deformity of the fingers in cerebral palsy can result from two reasons and it is a must to differentiate them in order to treat it appropriately. A swan neck deformity of the fingers with MCP flexion posture is because of intrinsic spasticity and the one with MCP extension posture is because of over activity of the long extensors. The former needs release of the spastic intrinsic muscle and the latter can get better with correction of the wrist flexion posture itself. Arthrodesis of the PIP joints in functional position in selected patients can give good results.

While doing correction of the spastic thumb we need to address four issues- release of the spastic adductor and flexor muscles; augmentation of the weak extensors and abductors; addressing the first web contracture and stabilization of the joints. A detailed assessment is needed to find out the contribution of each component and all need to be addressed at same time or else the result would be poor.

## Uncommon cases

Uncommon and rare presentations need more detailed analysis of the case and a tailor-made approach. Authors would present their experience in managing extensor spasticity of the hand and abduction and external rotation contracture of the shoulder.

## Unsolved problems

Presence of severe dystonia and athetosis remains a challenge and usually such patients cannot be made better by surgery.

It still remains a challenge to make these children use their hand even after a seemingly successful surgery because of the ‘learnt disuse’. Children also find it difficult to dissociate the involved limb from the movement of the contralateral limb and it is difficult of correct.

Authors try to extend the indications for surgery in patients who are disabled with severe bilateral deformity and are not using the hand for any activities. In such cases even if they are not “text book indications” we operate after a thorough analysis as for such cases – ‘any improvement is a gain and for someone who has nothing, a little is a lot’!

